# Visual Evaluation Strategies in Art Image Viewing: An Eye-Tracking Comparison of Art-Educated and Non-Art Participants

**DOI:** 10.3390/jemr19010014

**Published:** 2026-01-30

**Authors:** Adem Korkmaz, Sevinc Gülsecen, Grigor Mihaylov

**Affiliations:** 1 Department of Computer Technologies, Gonen Vocational School, Bandirma Onyedi Eylul University, Bandirma 10200, Türkiye; 2 Department of Computer Science, Istanbul University, Istanbul 34134, Türkiye; gulsecen@istanbul.edu.tr; 3 Department of Telecommunications, University of Telecommunications and Post, 1700 Sofia, Bulgaria; gregmihaylov@gmail.com

**Keywords:** tacit knowledge, human–computer interaction, screen-based eye tracking, visual perception, attention and cognition, eye tracking in education, digital assessment

## Abstract

Understanding how tacit knowledge embedded in visual materials is accessed and utilized during evaluation tasks remains a key challenge in human–computer interaction and visual expertise research. Although eye-tracking studies have identified systematic differences between experts and novices, findings remain inconsistent, particularly in art-related visual evaluation contexts. This study examines whether tacit aspects of visual evaluation can be inferred from gaze behavior by comparing individuals with and without formal art education. Visual evaluation was assessed using a structured, prompt-based task in which participants inspected artistic images and responded to items targeting specific visual elements. Eye movements were recorded using a screen-based eye-tracking system. Areas of Interest (AOIs) corresponding to correct-answer regions were defined a priori based on expert judgment and item prompts. Both AOI-level metrics (e.g., fixation count, mean, and total visit and gaze durations) and image-level metrics (e.g., fixation count, saccade count, and pupil size) were analyzed using appropriate parametric and non-parametric statistical tests. The results showed that participants with an art-education background produced more fixations within AOIs, exhibited longer mean and total AOI visit and gaze durations, and demonstrated lower saccade counts than participants without art education. These patterns indicate more systematic and goal-directed gaze behavior during visual evaluation, suggesting that formal art education may shape tacit visual evaluation strategies. The findings also highlight the potential of eye tracking as a methodological tool for studying expertise-related differences in visual evaluation processes.

## 1. Introduction

Information has historically been regarded as one of the most valuable forms of capital for individuals and institutions. Francis Bacon’s well-known statement, “Knowledge is power,” underscores how the effective use of knowledge can transform decision-making processes and individual actions [1]. However, acquiring and transferring knowledge directly is not always feasible. In *The Tacit Dimension*, Michael Polanyi [2] observes that “we can know more than we can tell,” emphasizing that substantial elements of human knowledge are not consciously accessible and cannot be articulated explicitly. Accordingly, tacit knowledge is commonly defined as knowledge shaped by individuals’ intuitions, experiences, and cognitive presuppositions [3].

Polanyi’s [2] conceptualization has been expanded through subsequent theoretical contributions. Kakabadse et al. [4] argue that tacit knowledge comprises both technical and cognitive components, whereas Nonaka and Takeuchi [3] emphasize that tacit knowledge is primarily grounded in technical skills. Because tacit knowledge is often conveyed through practice, behavior, and interaction rather than formal documentation, identifying and evaluating tacit knowledge remains methodologically challenging. In this respect, information systems represent a key technological infrastructure that supports knowledge sharing and learning processes, including knowledge that is difficult to codify [5].

Contemporary computing systems are increasingly developed to optimize human–computer interaction (HCI) by incorporating capabilities to analyze user behavior [6]. To infer user preferences and improve system experiences, researchers examine the interaction traces individuals leave behind. Advances in computing and data science have therefore enabled more robust approaches to understanding cognitive processes and decision-making mechanisms. In this context, eye tracking has gained prominence as a measurement method because it provides objective indicators of how individuals allocate visual attention when interacting with visual information [7].

The eyes are widely regarded as fundamental indicators of cognitive function, reflecting perception, attention, and emotional states. Eye movements provide observable correlates of visual attention and information processing, enabling the empirical examination of how individuals allocate attention during visual evaluation tasks [8,9]. Empirical evidence supports the centrality of visual information in human cognition; for example, Shepard [8] demonstrated that memory capacity for visual information substantially exceeds that for verbal information. Moreover, eye tracking is a valuable method for examining human behavior by capturing moment-to-moment visual attention and processing strategies [9]. With advances in precision and cost-effectiveness, eye-tracking technology is now widely used across disciplines such as psychology, medical diagnostics, marketing, and HCI to analyze responses to visual information and to improve user experience.

In the context of visual evaluation, eye movement metrics are not interpreted as direct measures of attention, but as behavioral indicators of underlying visual inspection strategies [7,9]. For example, fixation-related measures may reflect the depth of visual processing at task-relevant regions, whereas saccade-related measures can indicate the efficiency or dispersion of visual search. Accordingly, differences in these metrics are interpreted in relation to task demands and domain-specific experience rather than as general indicators of attentional capacity.

Despite these advances, operationalizing tacit knowledge during visual evaluation tasks remains methodologically demanding, as tacit processes are not directly observable through self-report. Eye tracking offers a promising route to address this challenge by providing quantitative metrics—such as fixation, visit, and gaze-related measures—that can reflect systematic differences in visual attention allocation and inspection strategies. Building on this rationale, the present study investigates whether tacit knowledge embedded in visual materials can be inferred in an HCI context using eye-tracking, comparing participants with formal art-education coursework (art-education group) to participants from non-art disciplines (non-art group).

In the present study, “visual evaluation” refers to a time-constrained, prompt-driven inspection task in which participants view art-history images and respond to item prompts targeting specific visual properties (e.g., composition, texture, perspective, and foreshortening). The task, therefore, does not involve free viewing; rather, it constitutes an accuracy-oriented evaluation process in which attention to prompt-relevant image regions is central. Because expert–novice differences in eye movement behavior are highly task-dependent, this task structure is critical for interpreting between-group differences in AOI-level and image-level metrics.

This study aims to examine between-group differences in eye-tracking metrics during the evaluation of art-history visual materials. The art-education group comprises undergraduate students with formal visual-arts training, including coursework involving structured visual analysis such as composition and form. In contrast, the non-art group consists of undergraduate students from non-art disciplines with no formal art-education coursework. The study addresses the following research questions: (1) Is there a statistically significant difference between art-education and non-art groups in AOI-based metrics within images? Moreover, (2) Is there a statistically significant difference between the art-education and non-art groups in overall image-evaluation processes as reflected by image-level eye movement measures?

Importantly, this study does not aim to estimate the causal effect of a single course. Instead, it compares visual evaluation strategies between participants with formal art-education coursework and participants from non-art disciplines using AOI-based and image-level eye-tracking metrics applied to standardized art-history stimuli. Accordingly, the findings are reported as between-group differences associated with educational background. They are intended to inform HCI-oriented interpretations of how visual training and experience relate to attention allocation during complex image evaluation.

## 2. Related Work

Eye tracking has become a prominent research tool for assessing cognitive processes, particularly in contexts where attention allocation and visual strategies provide indirect evidence of underlying cognition. DeLeeuw and Mayer [10] showed that multiple techniques can be used to evaluate cognitive processing during learning, and eye tracking has been widely recognized as a reliable approach for estimating cognitive load by analyzing how individuals distribute visual attention across learning materials [11].

A significant line of research uses eye tracking to differentiate experts from novices in tasks that require visual diagnosis, evaluation, or decision-making. In medical imaging, Jarodzka et al. [12] reported that experts attend more to task-relevant elements, employ knowledge-based shortcuts, and manage visual cognitive processing more efficiently. Similar expert–novice distinctions have been reported in other diagnostic contexts. For example, Warren et al. [13] found that expert veterinary pathologists reached cytology diagnoses both faster and with higher accuracy. In contrast, Dzeng et al. [14] showed that experienced construction workers identified hazards more quickly. Importantly, these findings indicate that performance differences across expertise levels may manifest in different dimensions—such as speed or accuracy—and that faster task completion does not necessarily imply higher accuracy.

In educational settings, eye tracking has also been used to examine how expertise shapes attention and monitoring strategies. Prior work has linked teachers’ gaze behavior to classroom management and the observation of student behavior [15,16,17]. Wolff et al. [15] reported that expert teachers direct their gaze more deliberately, whereas novice teachers exhibit more dispersed attention patterns. Extending this perspective, Seidel et al. [17] showed that expert teachers demonstrated stronger diagnostic skills when observing student engagement and were better able to identify individual needs.

Beyond formal education, eye-tracking has been used to understand skill-related differences in complex problem-solving tasks. Bednarik [18] analyzed programmers’ eye movements during debugging and found that experienced developers integrate information more effectively and adapt their visual strategies during the task. In multiple-choice problem solving, Tsai et al. [19] reported distinct attention patterns between successful and unsuccessful students, particularly in how they allocate gaze to relevant versus irrelevant factors. Complementing these findings, Lindner et al. [20] showed that learners with higher prior knowledge spent more time processing correct answers in multiple-choice testing.

Several studies further demonstrate how eye tracking can support training and performance enhancement by externalizing expert visual behavior. Jarodzka et al. [21] employed expert eye movement modeling examples (EMMEs) in medical education, using gaze guidance to help students focus on diagnostically relevant symptoms. Koury et al. [22] similarly reported that training materials incorporating expert gaze data may improve novices’ efficiency in leukocyte-identification tasks. Related work in reading and learning contexts indicates that stimulus characteristics may shape processing efficiency: Mason et al. [23] found that abstract images can facilitate more efficient text processing during online reading.

Eye tracking has also been used to examine domain-specific visual strategies in spatial and operational tasks. Dong et al. [24] reported that geographers outperformed non-geographers in map-reading accuracy, while Keil et al. [25] showed that reduced attention to off-route areas can support attentional control during route recognition. Beitlova et al. [26] found that geography teachers and students employ different scanning paths during map reading, and Keskin et al. [27] combined EEG and eye-tracking to evaluate cognitive load in expert versus novice map readers, showing that experts develop more effective visual strategies. In aviation, Ho et al. [28] observed that commercial pilots focus more on the primary flight display, whereas military pilots allocate more attention to external displays.

Finally, eye tracking has also been employed to infer latent cognitive states during testing and problem solving. Yamada et al. [29], for example, reported an accuracy of 90.1% in identifying students’ confidence levels on multiple-choice tests using gaze behavior. Similarly, Bi and Reid [30] found that successful solvers in static problem-solving tasks exhibited distinct gaze strategies compared with participants who failed to reach correct solutions.

A growing body of research has further demonstrated that art-related expertise systematically modulates eye movement behavior during the viewing and evaluation of visual material. Kołodziej et al. [31] showed that visual-art experts can be distinguished from non-experts based on oculomotor signatures, reflecting more structured and selective visual strategies. Likewise, Hu et al. [32] reported that art-trained observers exhibit different gaze distributions and esthetic evaluation patterns when judging visual icons. In the context of visual landscapes, Dupont et al. [33] demonstrated that domain-specific visual expertise alters how viewers allocate attention across complex scenes. More recently, Stein et al. [34] found that sculpture experts display more organized scan paths and longer dwell times in task-relevant regions than non-experts. Together, these studies indicate that formal visual-art training shapes esthetic judgments but also the underlying visual inspection strategies captured by eye-tracking metrics.

Taken together, this literature underscores that eye tracking is a powerful method for examining learning, decision making, and the development of visual expertise across diverse domains. Building on these findings, the present study examines whether differences in visual evaluation associated with educational background, specifically formal art education, can be detected using AOI-based and image-level eye-tracking metrics.

Despite the extensive evidence for expert–novice differences in gaze behavior, relatively few studies explicitly frame these differences as observable indicators of tacit knowledge during visual evaluation tasks, particularly using standardized AOI-based operationalizations that enable reproducible between-group comparisons. Moreover, prior research often focuses on domain-specific expertise without jointly reporting complementary AOI-level and image-level metrics within a single experimental paradigm. To address these gaps, the present study investigates whether differences in educational background can be captured through a combined set of AOI-based (fixation, visit, and gaze) and image-level (saccade behavior and pupil size) eye-tracking measures during the evaluation of visual materials.

## 3. Materials and Methods

This section details the methodology adopted in line with the study aim, including the experimental design, the study sample, the data collection instruments, and the data analysis procedure.

### 3.1. Research Model and Experimental Design

This study employed a quantitative, between-subjects comparative design using two naturally occurring groups defined by educational/disciplinary background: (i) an art-education group (Painting–Art Education program, including formal visual-arts training and structured visual analysis coursework) and (ii) a non-art group (Nursing program). Because group membership was not randomized and the groups differed in disciplinary background (and data were collected in two institutional settings), the findings are interpreted as associative between-group differences rather than as causal effects of a single course. The primary aim was to examine differences in eye-tracking metrics during visual evaluation tasks.

In line with this aim, participants’ visual perception processes were evaluated under controlled conditions to capture group-level differences in visual attention. Eye tracking was employed as the primary data collection method. By recording gaze direction and ocular movements, eye tracking enables the analysis of visual information processing in a structured laboratory setting [35]. All study procedures were conducted under standardized conditions, and the necessary ethical approvals were obtained prior to data collection [36].

### 3.2. Study Sample

Given the use of naturally occurring, non-randomized groups, causal inferences should be made cautiously; accordingly, the results are interpreted as between-group differences associated with educational and disciplinary background. A purposive sampling approach was used to determine the study sample [36,37]. The art-education group consisted of undergraduate students enrolled in the Painting–Art Education program within the Department of Fine Arts Education, Faculty of Education, Bursa Uludağ University, whereas the non-art group comprised undergraduate students from the Department of Nursing, Faculty of Health Sciences, Bandırma Onyedi Eylül University.

The art-education group included second-, third-, and fourth-year undergraduate students and, accordingly, participants in this group had received approximately 2 to 4 years of formal university-level training in visual arts, including coursework in drawing, composition, perspective, and visual analysis. In contrast, participants in the non-art group (nursing students) had not received any formal visual-arts coursework as part of their academic curriculum.

Initially, 132 participants volunteered to take part in the study. Prior to data collection, participants reporting visual impairments or neurological or psychiatric conditions that could affect eye-tracking performance were excluded. During data collection, some recordings were removed due to unsuccessful calibration or insufficient eye-tracking data quality (e.g., excessive signal loss). As a result, the final analytic sample consisted of 112 participants (87 female, 25 male), including 51 in the non-art group and 61 in the art-education group. Only recordings that met predefined data quality criteria were included in the analyses.

### 3.3. Data Collection Instruments

Data from participants in both the art-education and non-art groups were collected using the following instruments:

- Demographic Information Form: Developed to collect participants’ age, gender, department, and year of study, as well as information regarding their experience in the arts.
- Eye-Tracking Device and Software: The Tobii Pro X2-60 (Tobii Technology AB, Danderyd, Sweden) eye tracker and Tobii Pro Lab 1.145.28180 software were used to measure participants’ responses to visual stimuli. Fixations and saccades were identified using the default event-detection settings of Tobii Pro Lab. Specifically, saccades were defined using Tobii’s standard velocity-based classification algorithm, and event segmentation was determined by the software’s default thresholds for saccade velocity and amplitude. No additional custom thresholds were applied. All eye movement events were detected consistently across participants using the same settings.
- Achievement Test Visual Materials: A comprehensive eye-tracking achievement test was developed to evaluate visual perception skills and to examine cognitive processes relevant to formal visual-art education (e.g., composition, texture, perspective, and structured visual analysis). Visual stimuli were selected from prominent works in art history. Works by artists such as Leonardo da Vinci, Edgar Degas, Pablo Picasso, Raphael, M. C. Escher, and Sandro Botticelli were included to assess participants’ perception of light, texture, line types, perspective, foreshortening, triangular composition, and the characteristics of organic versus artificial objects. Test items were designed to direct attention to specific visual elements within each artwork, and participants were instructed to view each image for a predefined duration. Heatmaps, scanpaths, and predefined Areas of Interest (AOIs) were used to analyze saccades and fixation durations and to compare visual information processing between groups. Systematic examination of artistic works offers a practical approach for identifying attentional levels and differences in cognitive perception [38]. The test consisted of 9 images and 14 item prompts, developed by the researcher in consultation with domain experts. A pilot administration was conducted with 15 non-art and 15 art-education students, and the final version of the test was established based on this pilot.
- AOI definitions were developed in consultation with domain experts in visual arts education. In particular, the AOI mapping was created under the supervision of Prof. Dr. Ayhan Özer (Department of Painting, Faculty of Fine Arts, Gaziantep University), who held the title of Associate Professor at the time the AOIs were established.
- The visual stimuli consisted of well-known artworks selected from established art-history sources to represent a range of visual analysis targets relevant to the task, including composition, texture, perspective, foreshortening, and object characteristics. Stimulus selection was guided by instructional and analytic considerations rather than by an intention to elicit affective or emotional responses. Accordingly, the artworks were not formally normalized for participants’ prior familiarity or emotional valence.
- This design choice reflects the study’s focus on goal-directed, feature-based visual evaluation rather than affective engagement; that is, participants were instructed to locate task-defined visual targets (e.g., lines, textures, perspective cues) rather than to provide subjective esthetic judgments. To ensure comparability across participants despite the absence of formal normalization, all stimuli were presented under standardized viewing conditions, including identical display parameters, fixed presentation durations, and uniform task instructions. Furthermore, Areas of Interest (AOIs) were defined a priori based on item prompts and correct-answer regions, ensuring that all participants were evaluated with respect to the same task-relevant visual features.

### 3.4. Task and Trial Structure

Participants completed an eye-tracking–based visual achievement task comprising nine artworks and 14 item prompts, developed in consultation with domain experts and finalized following pilot administration. Each trial presented an artwork together with a corresponding prompt designed to direct attention to a specific target visual element within the image (e.g., line, texture, perspective, or compositional focus).

In the present study, some artworks were intentionally presented multiple times, each time accompanied by a different prompt targeting a distinct visual feature (e.g., light, texture, line orientation, depth, or compositional structure). These repeated presentations were part of a structured, feature-based task design rather than unintended stimulus repetition. All participants completed the tasks in the same predetermined sequence, which was designed to reflect a systematic progression commonly used in formal art education. Accordingly, any potential order or repetition effects were held constant across participants and therefore did not confound between-group comparisons.

The item prompts were not intended to represent graded difficulty levels, nor were they formally balanced with respect to question type or visual complexity. Instead, the task was designed to sample a range of core visual analysis concepts commonly addressed in formal art education curricula. Thus, variability across items reflects differences in targeted visual features rather than systematic manipulation of task difficulty.

Images were displayed for a fixed viewing duration (5 s or 10 s, depending on the item) under a standardized protocol. The assignment of 5 s versus 10 s exposure times was determined during the test development phase and specified a priori as part of the standardized visual achievement task. Shorter exposure times (5 s) were assigned to prompts targeting relatively localized or clearly identifiable visual elements (e.g., light areas, texture, specific line orientations, or profile views). In contrast, longer exposure times (10 s) were assigned to prompts that required integrating more global or structurally complex visual properties (e.g., depth cues, perspective organization, or spatial composition). These durations were fixed for each prompt and applied identically to all participants.

To enable comparable between-group analyses, Areas of Interest (AOIs) were defined a priori based on expert judgment and the target regions specified by the prompts. Accordingly, AOIs corresponded to the correct-answer elements within each artwork and were applied consistently across participants. Because AOIs represented task-defined target regions, gaze allocation within these AOIs served as an implicit indicator of task compliance, allowing verification that participants attended to the instructed visual elements even in the absence of overt behavioral responses.

AOIs were defined to tightly encompass the task-relevant visual targets specified by each prompt. Because different visual features (e.g., vanishing points, profiles, or compositional foci) vary in spatial extent, AOI size naturally varied across items. However, for any given stimulus, the same AOI was applied to all participants. Accordingly, group comparisons were conducted within identical AOIs for each item, ensuring that AOI size did not confound between-group differences in eye movement metrics.

### 3.5. Data Collection Procedure

Research environment and apparatus. Data were collected using a Dell Precision M6800 laptop (17.3-inch UltraSharp FHD display, 1920 × 1080) equipped with a Tobii Pro X2-60 eye tracker and Tobii Pro Lab software. Gaze data were recorded at 60 Hz, with an average visual angle accuracy of 0.5°. Data collection took place in sound- and light-controlled rooms allocated by the participating faculties.

Eye-tracking sessions were conducted in controlled laboratory environments. For the art-education group, data were collected in a dedicated office provided by the Department of Art Education (Painting–Art Teaching), Faculty of Education, Bursa Uludağ University; for the non-art group, an analogous environment was allocated by the Faculty of Health Sciences at Bandırma Onyedi Eylül University. Prior to testing, participants received detailed information about the study purpose and procedures, and written informed consent was obtained. After calibration, each participant completed an individual test session lasting approximately 8–12 min. The viewing distance was adjusted to approximately 50–70 cm. Although the viewing distance was not rigidly fixed, participants were seated in a stable position and instructed to minimize head movements. The Tobii Pro X2-60 eye tracker’s built-in head-movement tolerance and tracking algorithms were used to maintain the spatial accuracy of gaze-to-screen mapping throughout the trials. Each visual prompt was presented for a fixed duration (typically 5 s or 10 s, depending on the item), consistent with the standardized test protocol. All measurements followed standardized procedures designed to preserve the natural flow of eye movements.

The visual stimuli were presented centrally on the screen at a fixed size, preserving their original aspect ratios. Each image occupied approximately 70–80% of the 17.3-inch Full HD display area (1920 × 1080 pixels). Stimulus size and position were held constant across all trials and participants.

### 3.6. Data Analysis

Collected data were analyzed using Tobii Pro Lab v1.163 and SPSS 18.0. Normality was assessed using the Kolmogorov–Smirnov and Shapiro–Wilk tests. For normally distributed data, parametric tests, including the independent-samples *t*-test, were applied [39]. For non-normally distributed data, nonparametric tests, namely the Mann–Whitney U test, were used [40].

AOIs were defined a priori for each visual item based on expert judgment in the field and the intended target regions specified by the item prompts. The AOIs corresponded to the regions representing the correct-answer elements within each image and were created in Tobii Pro Lab prior to the primary analyses. The same AOI definitions were applied consistently across all participants to ensure comparability between groups. For each artwork/prompt pair, experts first identified the task-relevant visual element implied by the prompt and marked the corresponding correct-answer region on the stimulus. AOIs were then reviewed against the prompt wording to ensure one-to-one correspondence between the intended target feature and the AOI boundaries. Final AOIs were implemented in Tobii Pro Lab and applied identically across all participants.

Although the present study did not segment trials into discrete temporal phases (e.g., early vs. late interest periods), task dynamics were examined indirectly through time-sensitive eye-tracking metrics, including time to first fixation, first visit duration, mean visit duration, and total gaze duration at both AOI and image levels. These measures are commonly used to capture differences between initial orienting responses and more sustained, goal-directed inspection processes during visual evaluation. Trial durations varied across items (5 s or 10 s) based on the complexity of the target visual element; however, all participants viewed identical stimuli under the same timing conditions. Between-group comparisons, therefore, relied on relative differences in gaze allocation patterns rather than on absolute exposure time, reducing the risk that duration differences would confound group-level effects.

The eye-tracking metrics used in this study to evaluate participants’ cognitive attentional processes comprised the following core components:

- Fixation Duration: The amount of time a participant remains within a specific area of interest (AOI) [41].
- Visit Count: The number of times a participant enters a predefined AOI.
- Gaze Count: The total number of gaze events recorded within a given AOI.
- Saccade Amplitude: A measure of the magnitude of eye movements when transitioning from one AOI to another.
- Mean fixation duration was defined as the average duration of individual fixations within a given area of interest (AOI) or image, calculated by dividing the total fixation duration by the number of fixations recorded in that region during the viewing period.

These metrics were used to examine how participants perceived the visual materials and which regions they prioritized during information processing [42,43]. Overall, the resulting dataset provides detailed evidence for understanding participants’ visual information processing.

No additional behavioral measures (e.g., response accuracy scores, reaction times, or explicit performance ratings) were analyzed beyond eye movement data. The task was designed as a structured visual evaluation intended to elicit differences in visual inspection strategies rather than to assess overt behavioral performance. Accordingly, eye-tracking metrics served as the primary indicators of task-related visual processing.

The selection of eye movement indices in the present study was guided by their established associations with different aspects of visual inspection and information processing. Fixation count and fixation-related duration measures (e.g., mean and total gaze and visit durations within AOIs) were examined as indicators of sustained visual engagement with task-relevant image regions. Higher values in these measures suggest more prolonged and focused visual processing rather than superficial scanning.

Saccade-related measures (e.g., saccade count) were included to characterize global patterns of visual exploration across the image. A higher saccade count is commonly interpreted as reflecting a broader, less selective scanning strategy. In contrast, a lower saccade count may indicate more systematic inspection when task-relevant regions are efficiently prioritized. Image-level metrics were therefore used to capture overall viewing behavior, while AOI-based metrics targeted attention allocation to predefined, task-relevant regions. Importantly, these indices are not treated as direct measures of visual attention per se, but as observable correlates of visual processing strategies under the constraints of the evaluation task.

## 4. Results

This section reports the analyses of data obtained from the eye-tracking achievement test and the personal information form for the non-art group (Nursing students) and the art-education group (Painting–Art Education students with formal visual-arts training). The data were evaluated using Tobii Pro Lab and SPSS 18.0, and the significance level was set at *p* = 0.05.

The eye-tracking results are organized into two complementary levels of analysis. First, AOI-based eye movement behavior is reported, reflecting participants’ visual engagement with task-relevant target regions specified by the prompts. Second, image-level eye movement behavior is reported, capturing overall viewing patterns across the entire artwork that are not constrained to predefined AOIs. This structure allows the examination of both targeted, task-driven attention and more global viewing behavior during image evaluation.

### 4.1. AOI-Based Eye Movement Behavior

To determine whether the AOI-related eye-tracking data met the normality assumption, the Kolmogorov–Smirnov test was applied. The results indicated that the data were normally distributed (*p* > 0.05); therefore, independent-samples *t*-tests were conducted. As shown in Table 1, there was a statistically significant difference between groups in AOI fixation count (t(110) = 4.81, *p* < 0.05). The Art-education group demonstrated a higher mean fixation count (M = 5.95) than the non-art group (M = 4.74), suggesting that participants in the art-education group fixated more on the correct answer.

In contrast, no statistically significant difference was found between groups in AOI visit count (t(110) = 1.57, *p* > 0.05). This indicates that the frequency of AOI entry did not differ meaningfully between the art-education and non-art groups. Accordingly, the findings suggest that fixation count is a more salient factor than visit count in distinguishing performance related to identifying the correct answer.

To compare the non-art and art-education groups on time to first fixation, first-visit duration, and first-gaze duration, the Kolmogorov–Smirnov test was used to assess normality. Time to first fixation did not follow a normal distribution (*p* < 0.05); therefore, the Mann–Whitney U test was applied. Because first visit duration and first gaze duration were normally distributed (*p* > 0.05), independent-samples t-tests were conducted.

As shown in Table 2, no statistically significant difference was found between groups in time to first fixation (U = 1374.50, *p* > 0.05), indicating that time to first fixation was not a discriminative factor between groups. In contrast, first visit duration differed significantly between groups (t(110) = 4.58, *p* < 0.05), with a higher mean in the Art-education group (M = 709.38 ms) than in the non-art group (M = 470.28 ms). Similarly, first gaze duration also showed a statistically significant between-group difference (t(110) = 4.54, *p* < 0.05), with the Art-education group exhibiting a higher mean (M = 731.39 ms) than the non-art group (M = 493.21 ms). Overall, these results suggest that first-visit duration and first-gaze duration contribute to identifying the correct answer. In contrast, time to first fixation does not appear to be a determining factor.

Eye-tracking data for AOI mean fixation duration, mean visit duration, and mean gaze duration were first examined using normality tests. Because mean fixation duration was normally distributed (*p* > 0.05), an independent-samples *t*-test was applied. However, mean visit duration and mean gaze duration were not normally distributed (*p* < 0.05); therefore, Mann–Whitney U tests were used.

As reported in Table 3, no statistically significant difference was observed between the art-education and non-art groups in mean fixation duration (t(110) = 0.049, *p* > 0.05), indicating comparable performance in average fixation time within the AOI. In contrast, statistically significant between-group differences were identified for both mean visit duration and mean gaze duration. For mean visit duration, the art-education group had a higher mean rank (65.23) than the non-art group (46.06) (U = 1023.00, *p* < 0.05), indicating that the art-education group spent more time per visit within the AOI. Similarly, for mean gaze duration, the art-education group also exhibited a higher mean rank (64.90) than the non-art group (46.45) (U = 1043.00, *p* < 0.05), indicating longer gaze allocation to the AOI. Overall, these findings suggest that mean visit duration and mean gaze duration are informative for detecting or prioritizing the AOI. In contrast, mean fixation duration does not yield a discriminative difference between groups.

Independent-samples t-tests were conducted to compare the art-education and non-art groups on total fixation duration, total visit duration, and total gaze duration. As shown in Table 4, a statistically significant difference was found between groups for total fixation duration (t(110) = 3.44, *p* < 0.05). The art-education group exhibited a higher mean total fixation duration (M = 1897.36 ms) than the non-art group (M = 1558.63 ms), indicating that participants in the Art-education maintained by the group fixation on the AOI for longer overall.

Similarly, a significant between-group difference was observed for total visit duration (t(110) = 4.18, *p* < 0.05), with the Art-education group showing a higher mean (M = 2021.51 ms) than the non-art group (M = 1613.67 ms). In addition, total gaze duration was also significantly higher in the art-education group (M = 2117.91 ms) than in the non-art group (M = 1709.35 ms) (t(110) = 4.08, *p* < 0.05). Collectively, these results indicate that the art-education group demonstrated higher overall engagement with the AOI in terms of total fixation, visit, and gaze durations, consistent with background-related differences in attention allocation during image evaluation.

Between-group differences in maximum fixation duration, maximum visit duration, and maximum gaze duration were examined using independent-samples *t*-tests. As reported in Table 5, no statistically significant difference was observed between groups for maximum fixation duration (t(110) = 1.43, *p* > 0.05), indicating that maximum fixation duration is not a discriminative factor for detecting the AOI.

In contrast, the maximum visit duration was significantly higher in the art-education group (M = 1063.77 ms) than in the non-art group (M = 761.52 ms) (t(110) = 5.36, *p* < 0.05). Likewise, the maximum gaze duration was also higher in the Art-education group (M = 1089.07 ms) than in the non-art group (M = 785.57 ms) (t(110) = 5.36, *p* < 0.05). Overall, these results suggest that participants in the art-education group achieved higher performance in maximum visit and gaze durations, supporting the interpretation that these differences may reflect structured visual analysis practices associated with an art-education background.

### 4.2. Image-Level Eye Movement Behavior (AOI-Independent)

As reported in Table 6, the results indicate statistically significant differences between the art-education and non-art groups in fixation and saccade counts during image identification. According to the independent-samples *t*-test, the non-art group exhibited a higher mean fixation count (M = 15.05) than the art-education group (M = 14.04). This difference was statistically significant (t(110) = 2.03, *p* < 0.05). This finding suggests that participants in the art-education group produced fewer fixations than those in the non-art group while evaluating the images.

In addition, the Mann–Whitney U test showed that the Art-education group had a lower mean rank than the non-art group for saccade count (U = 1127.00, *p* < 0.05). Specifically, the non-art group’s mean rank (64.90) exceeded that of the art-education group (49.48), indicating that participants in the art-education group made fewer saccades across the images. Taken together, these findings suggest that the art-education group scanned the images more systematically, exhibited fewer eye movements overall than the non-art group, and adopted a more focused visual inspection strategy during item evaluation.

As shown in Table 7, no statistically significant differences were observed between the art-education and non-art groups for time to first fixation (U = 1365.00, *p* > 0.05) or mean fixation duration (U = 1412.50, *p* > 0.05). This indicates that participants’ initial and average fixation durations during the evaluation of the visual items were not discriminative factors in the process of identifying the correct answer.

In contrast, a statistically significant difference between groups was found in total fixation duration (U = 1016.00, *p* < 0.05). Based on the mean ranks, the non-art group had a higher total fixation duration. In contrast, the art-education group had a lower overall fixation time across the images. These results suggest that total fixation duration is a meaningful factor in identifying the correct answer. In contrast, time to first fixation and mean fixation duration do not appear to play a determining role.

As shown in Table 8, the independent-samples t-test indicated no statistically significant difference between the art-education and non-art groups in mean pupil size during the process of identifying the correct answer in the images (t(110) = 1.04, *p* > 0.05). Although the art-education group exhibited a slightly higher mean pupil size (M = 3.85) than the non-art group (M = 3.75), this difference was not statistically significant. These results suggest that pupil size was not a determining factor during item evaluation and did not yield a meaningful between-group difference in attentional engagement.

Mean and total saccade amplitude during participants’ evaluation of the images were analyzed using the Mann–Whitney U test. As shown in Table 9, no statistically significant difference was observed between the art-education and non-art groups in mean saccade amplitude (U = 1537.50, *p* > 0.05). This finding indicates that mean saccade amplitude was not a discriminative factor in the image-evaluation process.

In contrast, a statistically significant between-group difference was found for total saccade amplitude (U = 1206.00, *p* < 0.05). The non-art group exhibited higher total saccade amplitude than the art-education group, suggesting that participants in the Art-education group displayed more controlled eye movements by making fewer or less extensive saccades across the images. Accordingly, these results imply that total saccade amplitude is a more informative factor than mean saccade amplitude in the process of identifying the correct answer.

The highlighted region represents the task-relevant target area defined a priori based on expert judgment and the item prompt (e.g., foreshortening/rakursi). This AOI was used consistently across all participants for AOI-based analyses.

**Figure 1:**
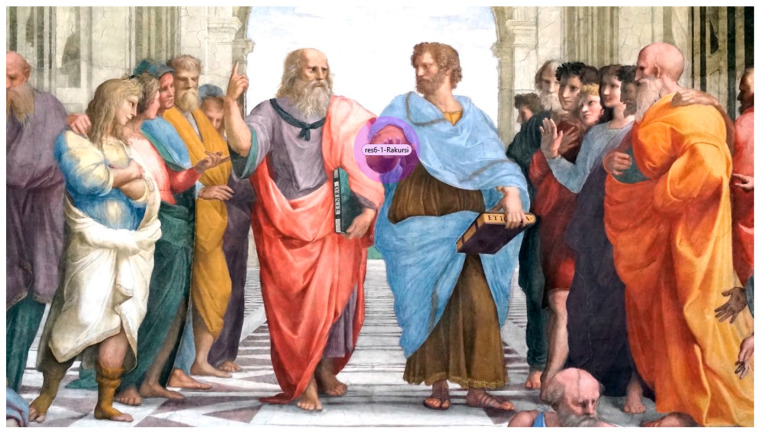
Example of a stimulus with predefined Areas of Interest (AOIs).

**Figure 2:**
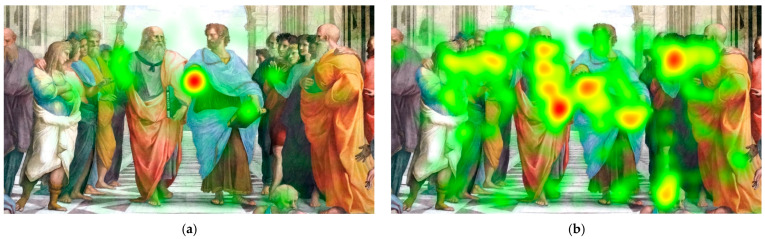
Group-level gaze heat maps for the task-relevant recursive point of interest: (**a**) art-education group and (**b**) non-art group..

As illustrated in Figure 1 and Figure 2, the predefined AOI corresponds to the task-relevant target region specified in the prompt (e.g., foreshortening/rakursi). Visual inspection of the group-level heatmaps reveals a clear between-group difference in gaze allocation. The art-education group (Figure 2a) showed a pronounced concentration of fixations within the AOI, indicating focused, selective visual processing of the target feature. In contrast, the non-art group (Figure 2b) showed a more diffuse gaze distribution across the image, with less spatial concentration on the AOI. This pattern is consistent with the AOI-based statistical results, supporting the interpretation that formal visual training is associated with more targeted and systematic visual evaluation strategies.

## 5. Conclusions and Discussion

From a process perspective, the observed between-group differences suggest that visual evaluation unfolds differently as a function of formal visual training. Specifically, the combination of fewer saccades and longer AOI visit durations in the art-education group indicates a shift from broad exploratory scanning toward sustained, goal-directed inspection of task-relevant regions. In this context, a “systematic” viewing strategy does not refer to spatial centering per se, but to the temporal stability and selectivity of gaze within the predefined AOI specified by the task. By contrast, a more spatially distributed gaze pattern characterized by higher saccade rates and shorter AOI engagement reflects exploratory rather than task-focused visual processing. Even in the absence of explicitly defined interest periods, these complementary gaze metrics capture early orienting and later verification stages of visual evaluation.

In this study, we examined how participants with and without formal art education differed in their image-evaluation processes using a comprehensive set of eye-tracking metrics. The findings revealed consistent between-group differences that reflect distinct visual evaluation strategies associated with educational background.

These differences should be interpreted in light of the task structure. The experimental paradigm required time-limited, prompt-driven evaluation of specific visual features rather than unconstrained esthetic viewing. Previous research has shown that expert–novice differences in gaze behavior are influenced by task demands, such as free viewing, feature search, or diagnostic judgment. Accordingly, the higher AOI engagement and reduced saccadic activity observed in the art-education group should be understood as differences in strategy under goal-directed and target-specified viewing conditions.

At the AOI level, the art-education group exhibited significantly higher fixation counts on the correct-answer regions, indicating a greater allocation of visual processing to task-relevant image elements. Although no between-group differences were observed for visit count or time to first fixation, the art-education group showed significantly longer first-visit and first-gaze durations upon reaching the correct regions, suggesting more intensive early-stage processing of task-relevant visual information.

Although the mean fixation duration did not differ significantly between groups, the art-education group showed a significantly higher mean visit duration and mean gaze duration. This pattern indicates that individual fixations were of similar temporal length across groups, but that art-educated participants engaged in more sustained and repeated inspection of task-relevant regions. Consistently, total fixation, total visit, and total gaze durations were all significantly higher in the art-education group, reflecting greater cumulative engagement with the instructed visual targets.

Similarly, although no group differences were observed for maximum fixation duration, the art-education group exhibited significantly higher maximum visit and maximum gaze durations. These findings indicate that art-educated participants were more likely to sustain prolonged episodes of focused inspection on specific areas of interest, consistent with more profound and more systematic visual analysis.

At the image level, the non-art group showed higher saccade and fixation counts, suggesting broader and more dispersed scanning patterns. In contrast, the art-education group exhibited fewer saccades, consistent with more selective, targeted exploration of the visual material. Mean pupil size did not differ significantly between groups, indicating that the observed gaze differences were unlikely to be driven by general differences in cognitive load.

The present findings are broadly consistent with prior work, although the literature shows variability depending on task characteristics. While some studies have reported longer fixation durations among experts or art-educated observers [44,45,46,47,48], others have found fewer fixations and shorter fixation durations in expert groups [32,49,50], highlighting the task-dependent nature of expertise effects. For example, Durugbo [51] reported that experts produced more fixations on distractors when task demands required additional information processing. Similarly, Seya et al. [47] found fewer saccades but longer fixation durations in art-educated observers, a pattern that closely parallels the present results.

Comparable differences have also been documented in studies contrasting individuals with and without art education [31,32], supporting the view that visual perception and eye movement behavior change as a function of training and experience. More recent research further demonstrates that visual expertise is characterized by selective, goal-directed attention in domain-specific and authentic task contexts. For example, Stein et al. [34] showed that sculpture experts exhibit more structured scan paths and longer dwell times in task-relevant regions. Similar effects have been reported in applied domains such as sports and disaster inspection [52,53]. In line with this literature, the present study found that participants with formal art education displayed fewer saccades and longer cumulative gaze durations within task-relevant AOIs, reflecting more systematic and efficient visual evaluation.

Beyond these statistical differences, the findings have important conceptual and practical implications. At a theoretical level, they suggest that formal visual training is associated with qualitatively different visual evaluation strategies under goal-directed conditions. Rather than relying on broadly distributed scanning, art-educated participants appear to prioritize structurally and conceptually relevant regions of the image, allocating visual processing resources more selectively and efficiently.

From an applied perspective, these results are directly relevant to human–computer interaction contexts in which visual inspection, analysis, or decision making is central. Eye-tracking metrics may provide a non-intrusive means of distinguishing novice-like and expert-like visual strategies in educational, training, and assessment environments. For example, gaze-based indicators could be used to support adaptive learning systems, inform the design of visual training interfaces, or provide formative feedback on visual analysis skills without requiring explicit verbal reports. Importantly, eye movement measures are not treated as direct equivalents of visual attention, but rather as observable behavioral correlates of underlying visual processing strategies shaped by task demands, experience, and contextual constraints.

An important consideration is whether the observed between-group differences reflect visual evaluation strategies rather than differential interest in artworks per se. Several aspects of the experimental design argue against a pure interest-based interpretation. First, the task was prompt-driven and goal-directed, requiring participants to locate specific visual features rather than to engage in free viewing or esthetic appraisal. Second, AOIs were defined based on task-relevant target regions rather than on visually salient or esthetically prominent areas. Third, comparisons were conducted between groups viewing identical stimuli under identical conditions, such that any general interest in the artworks would be expected to affect both groups similarly. Together, these design features support the interpretation that the observed differences primarily reflect differences in visual evaluation strategies shaped by formal training, rather than differences in intrinsic interest in the artworks.

### Limitations

This study has several limitations. First, it used a quasi-art-education, between-subjects design with naturally occurring (non-randomized) groups; therefore, the findings should be interpreted as between-group differences associated with educational/disciplinary background rather than causal effects. Second, the art-education and non-art groups were drawn from different academic programs (Painting–Art Education vs. Nursing), potentially introducing confounding due to disciplinary backgrounds (e.g., baseline visual literacy, familiarity with art-based stimuli, and motivation). Third, although AOIs were defined a priori based on expert judgment and item prompts to standardize analyses, AOI specification may influence metric estimates. Fourth, multiple metrics were tested across several comparisons, which may increase the risk of Type I error. Finally, the sample comprised undergraduate students from two institutions, and data were collected using a single screen-based eye-tracking setup in a controlled laboratory context; thus, generalizability to other populations and more ecologically valid HCI settings may be limited.

A limitation of the present study is that the visual stimuli were not normalized for prior familiarity or emotional valence. Although all participants viewed the same artworks under identical conditions, individual differences in prior exposure or affective response may have influenced gaze behavior. Future studies could address this limitation by pre-assessing familiarity and emotional valence ratings or by employing controlled stimulus sets with established normative values.

Future research could extend the present approach by explicitly segmenting trials into interest periods (e.g., early orientation versus later verification phases) or by normalizing gaze metrics across time windows, thereby further refining the characterization of visual evaluation dynamics.

Whether similar differences would emerge with non-art visual stimuli (e.g., technical diagrams or natural scenes) cannot be determined from the present data. However, the methodological framework, prompt-driven tasks combined with AOI-based eye-tracking analysis, can be readily applied to other visual domains, providing a basis for future research on the generalizability of these findings beyond art-related stimuli.

## 6. Recommendations

This study demonstrates that individuals in the art-education group and the non-art group follow different visual evaluation processes, as evidenced by eye-tracking data. The findings indicate that individuals in the art-education group exhibit more systematic attentional processing toward AOIs and sustain attention for longer when evaluating images.

Based on these results, the following recommendations are proposed for future research:

- Future studies may integrate eye-tracking metrics from individuals in the art-education group and the non-art group with machine learning methods to develop classification models and to examine how visual evaluation strategies evolve across educational levels.
- Eye-tracking studies can be conducted with groups differing in age and experience to determine how visual materials may be integrated into educational processes.
- To examine visual learning and cognitive processes, eye-tracking data can be analyzed using machine learning methods to develop classification models.
- Differences between individuals with and without art education can be investigated more comprehensively to understand better factors shaping artistic perception.
- Longitudinal analyses can be conducted across different educational levels to evaluate how visual attention processes evolve as experience is gained.

Overall, this study indicates that eye tracking is an effective tool for identifying cognitive differences within educational contexts and for revealing how visual scanning strategies vary as a function of training level. Future research may extend these analyses by applying similar methods across domains and examining eye-tracking metrics in broader, more diverse samples.

## Figures and Tables

**Table 1:** Independent-samples t-test results for AOI fixation and visit counts by group.

Measure	Group	N	M	SD	df	t	*p*
Fixation count	Non-art	51	4.74	0.99	110	4.81	0.001
	Art-education	61	5.95	1.54			
Visit count	Non-art	51	3.30	0.76	110	1.57	0.118
	Art-education	61	3.53	0.80			

**Table 2:** Mann–Whitney U and independent-samples t-test results for AOI time to first fixation, first visit duration, and first gaze duration by group.

**Measure**	**Group**	**N**	**Mean Rank**	**Rank Sum**	**U**	***p***	
Time to first fixation	Non-art	51	52.95	2700.50	1374.50	0.290	
	Art-education	61	59.47	3627.50			
	**Group**	**N**	**M**	**SD**	**df**	**t**	***p***
First visit duration	Non-art	51	470.28	191.07	110	4.58	0.001
	Art-education	61	709.38	328.40			
First gaze duration	Non-art	51	493.21	192.49	110	4.54	0.001
	Art-education	61	731.39	329.87			

**Table 3:** Independent-samples t-test and Mann–Whitney U test results for AOI mean fixation duration, mean visit duration, and mean gaze duration by group.

**Measure**	**Group**	**N**	**M**	**SD**	**df**	**t**	***p***
Mean fixation duration	Non-art	51	838.68	271.66	110	0.049	0.961
	Art-education	61	841.34	302.17			
	**Group**	**N**	**Mean rank**	**Rank sum**	**U**	***p***	
Mean visit duration	Non-art	51	46.06	2349.00	1023.00	0.002	
	Art-education	61	65.23	3979.00			
Mean gaze duration	Non-art	51	46.45	2369.00	1043.00	0.003	
	Art-education	61	64.90	3959.00			

**Table 4:** Independent-samples t-test results for AOI total fixation duration, total visit duration, and total gaze duration by group.

Measure	Group	N	M	SD	df	t	*p*
Total fixation duration	Non-art	51	1558.63	425.68	110	3.44	0.001
	Art-education	61	1897.36	585.12			
Total visit duration	Non-art	51	1613.67	434.60	110	4.18	0.001
	Art-education	61	2021.51	570.47			
Total gaze duration	Non-art	51	1709.35	446.85	110	4.08	0.001
	Art-education	61	2117.91	585.20			

**Table 5:** Independent-samples t-test results for AOI maximum fixation duration, maximum visit duration, and maximum gaze duration by group.

Measure	Group	N	M	SD	df	t	*p*
Maximum fixation duration	Non-art	51	1019.90	323.01	110	1.43	0.154
	Art-education	61	1122.58	417.06			
Maximum visit duration	Non-art	51	761.52	222.51	110	5.36	0.001
	Art-education	61	1063.77	347.09			
Maximum gaze duration	Non-art	51	785.57	223.72	110	5.36	0.001
	Art-education	61	1089.07	348.60			

**Table 6:** Mann–Whitney U and independent-samples t-test results for saccade count and fixation count by group.

**Measure**	**Group**	**N**	**M**	**SD**	**df**	**t**	***p***
Fixation count	Non-art	51	15.05	2.09	110	2.03	0.044
	Art-education	61	14.04	2.98			
	**Group**	**N**	**Mean rank**	**Rank sum**	**U**	***p***	
Saccade count	Non-art	51	64.90	3310.00	1127.00	0.012	
	Art-education	61	49.48	3018.00			

**Table 7:** Mann–Whitney U test results for time to first fixation, mean fixation duration, and total fixation duration across images by group.

Measure	Group	N	Mean Rank	Rank Sum	U	*p*
Time to first fixation	Non-art	51	60.24	3072.00	1365.00	0.266
	Art-education	61	53.38	3256.00		
Mean fixation duration	Non-art	51	59.30	3024.50	1412.50	0.403
	Art-education	61	54.16	3303.50		
Total fixation duration	Non-art	51	67.08	3421.00	1016.00	0.002
	Art-education	61	47.66	2907.00		

**Table 8:** Independent-samples *t*-test results for mean pupil size across images by group.

Measure	Group	N	M	SD	df	t	*p*
Mean pupil size	Non-art	51	3.75	0.44	110	1.04	0.296
	Art-education	61	3.85	0.48			

**Table 9:** Mann–Whitney U test results for mean and total saccade amplitude across images by group.

Measure	Group	N	Mean Rank	Rank Sum	U	*p*
Mean saccade amplitude	Non-art	51	56.15	2863.50	1537.50	0.916
	Art-education	61	56.80	3464.50		
Total saccade amplitude	Non-art	51	63.35	3231.00	1206.00	0.041
	Art-education	61	50.77	3097.00		

## Data Availability

The data presented in this study are available on request from the corresponding author. The data are not publicly available due to privacy and ethical restrictions.
